# Concerning Auricular Vagal Nerve Stimulation: Occult Neural Networks

**DOI:** 10.3389/fnhum.2019.00421

**Published:** 2019-12-12

**Authors:** Yusuf Ozgur Cakmak

**Affiliations:** ^1^Department of Anatomy, School of Biomedical Sciences, University of Otago, Dunedin, New Zealand; ^2^Brain Health Research Centre, University of Otago, Dunedin, New Zealand; ^3^Centre for Health Systems and Technologies, University of Otago, Dunedin, New Zealand; ^4^Medical Technologies Centre of Research Excellence, Auckland, New Zealand

**Keywords:** auricular, vagus, electrostimulation, nVNS, facial nerve, neuromodulation, network, neuroanatomy

## Abstract

Auricular vagal nerve stimulation (AVNS) is an evolving neuromodulation technology that has a wide range of therapeutic applications across multiple disciplines of medical science. To date, AVNS results had been interpreted in the context of a monolog concept of the auricular branch of the vagus nerve (ABVN): that this is the sole network of the mechanism of action and/or structure in the auricular area of the stimulation in the context of activations in the brainstem nuclei, including the nucleus tractus solitarius (NTS), locus coeruleus (LC), trigeminal brainstem nuclei, and the nucleus cuneatus. This review considers the overlooked aspects of neural networks, connections, hijacking axons from cranial nerves and cervical sympathetic ganglions, the inhomogeneous distribution of perivascular sympathetic nerves, and intrinsic/extrinsic auricular muscles in the auricular zone that can explain the vagal and non-vagal nucleus activations in AVNS. In addition, the unique cortical representation of the human ear and interspecies differences in the auricula are discussed. The detailed auricular anatomy of the AVNS zone explored in the present study references structural and functional neural network information to overcome default designs and misinterpretations of existing research on AVNS to provide a better foundation for future investigations that use this modality.

## Introduction

The number of auricular vagal nerve stimulation (AVNS) studies in the literature has increased logarithmically over the last two decades (Cakmak, 2006; Badran et al., 2018a,b; Burger et al., 2019; Hong et al., 2019; Zhao et al., 2019). A broad range of clinical conditions across multiple disciplines—from neurology to immunology—have been investigated for a potential clinical therapeutic response to AVNS. However, most of these studies are designed and interpreted according to the same neuroanatomical model defined by retrograde neuronal tract tracing research, which is limited in terms of denervation levels as well as in the selection of the anatomical structures to be examined. In addition, functional magnetic resonance imaging (fMRI) studies designed to demonstrate the AVNS activations of the influenced networks and structures in response to non-invasive vagal nerve stimulation are also based on the same concept of AVNS neural connectivity and might therefore have overlooked other neural connections and structures in the zone of the AVNS. Multiple lines of evidence in the literature indicate that anatomical structures in the anatomical zone of AVNS can modulate the same neural vagal and non-vagal structures in the brainstem *via* alternative pathways. The inhomogeneous distribution of the nerves, including the perivascular sympathetic nerves, were not considered by these studies. It is also worth noting that the field electrical stimulation used in studies of the auricular branch of the vagus nerve (ABVN) cannot target a specific axon or nerve bundle in the stimulation zone and instead may modulate all the nerves and structures beneath and between the electrodes. In addition, the potential neural networks that can be manipulated as a result of such stimulation are beyond the monosynaptic anatomical connectivity that is described by neural tract tracing studies; multi-synaptic influences should always be considered to gain a more complete understanding of the potential of AVNS.

The aim of this article is to identify and highlight the anatomical aspects of auricular vagal stimulation zones overlooked by prior studies to yield an improved proxy to inform future research design and the interpretation of AVNS studies.

## The Path: Connectivity and Hijacker (Hitchhiker) Axons

### Jugular Foramen to Mastoid

**Figure 1:**
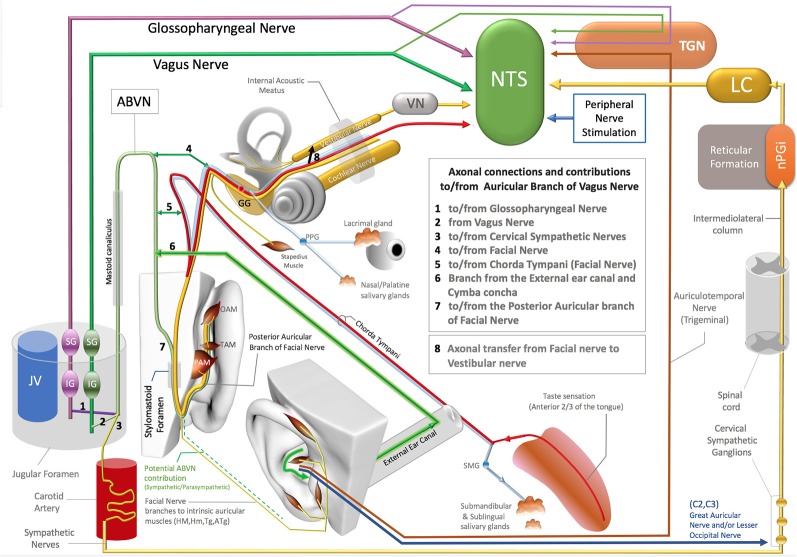
Axonal connections and contibutions to/from the auricular branch of the vagus nerve (ABVN), Non-vagal pathways to nucleus tractus solitarius (NTS), locus coeruleus (LC). TGN, Trigeminal sensory nuclei; nPGI, NucleusParagigantocellularis; VN, Vestibular Nuclei; GG, Geniculate ganglion of Facial Nerve; PPG, Pterygopalatine ganglion of the Facial Nerve; SMG, Submandibular ganglion of the Facial Nerve; OAM, Oblique auricular muscle; TAM, Transverse Auricular Muscle; PAM, Posterior Auricular Muscle; JV, Jugular Vein; IG (magenta), Inferior ganglion of the Glossopharyngeus nerve; SG (magenta), Superior ganglion of the glossopharyngeal nerve; SG (green), Superior Ganglion of the Vagus nerve; IG (Green), Inferior ganglion of the Vagus Nerve.

The ABVN pathway is quite complex on account the contribution of multiple axons to this branch along its path (Figure 1). The ABVN is known to originate in the superior (also referred to as the jugular) ganglion of the vagus nerve. However, from a functional anatomical perspective, it is more correct to take the sensory content of the axons into consideration and state vice versa. In this context, it is worth noting that only a portion of ABVN axons join with the superior vagal ganglion at the level of the jugular foramen; there are other non-vagal axons that leave the main branch of the ABVN at this same level. In other words, the ABVN axons split into three main bundles at the level of the jugular foramen (after it leaves the mastoid foramen-temporal bone and before terminating in the superior ganglion of the vagus nerve): (1) one bundle joins the glossopharyngeal nerve (Ozveren et al., 2003; Tubbs et al., 2005; Watanabe et al., 2016); (2) another axonal bundle (sympathetic nerves) joins the cervical sympathetic ganglions (that project through the internal carotid nerve, Matthews and Robinson, 2014); and (3) other axons terminate in the superior vagal ganglion. It is worth noting that the non-vagal bundles (1 and 2) branch off after the ABVN leaves the mastoid canaliculus and before the ABVN terminates in the superior vagal ganglion (Figure 1).

The most proximal ABVN injections in neural tract-tracing studies of the ABVN have been performed with injections of horseradish peroxidase (HRV) into the cut end (proximal to the facial nerve connection) of the ABVN before it splits into the non-vagal branches of the glossopharyngeal nerve and cervical sympathetic ganglions. Neural labeling has been demonstrated in the superior vagal ganglion, but not in the other cranial nerve ganglia, including the superior/inferior ganglion of the glossopharyngeal nerve, geniculate ganglion of the facial nerve, inferior (nodose) ganglion of the vagus nerve, and trigeminal ganglion (Nomura and Mizuno, 1984). In this context, the superior vagal ganglion has been proposed as the terminal for ABVN axons. On the other hand, the same study labeled fibers in the spinal trigeminal tract and the principal trigeminal nucleus, nucleus tractus solitarius (NTS), cuneate nucleus, and C1–3 dorsal horn segments after the same HRV injection (Nomura and Mizuno, 1984). Considering that most of these anatomical regions are non-vagal centers, a significant implication of this 1984 report has been overlooked: the authors failed to investigate the sympathetic cervical ganglions for potential labeling, and the cervical sympathetic ganglions are likely to be labeled with retrograde tracer injections into the same ABVN segment. In addition, all the reported labeling achieved by this same study, including the spinal trigeminal tract, principal trigeminal nucleus, NTS, cuneate nucleus, and C1–3 dorsal horns of the cervical spinal nerves after HRP injection into the ABVN segment situated distal to the non-vagal contributions to ABVN, cannot be explained by vagal axons. These are likely to be the axons that do not synapse in the superior vagal ganglia but exist in or hitchhike the ABVN. The auricular muscles are modulated along with the neck and shoulder muscles by neurons in the cuneate nucleus (Maslany et al., 1991); hence, the cuneate nucleus is likely to be excited with intrinsic auricular muscle stimulation in the ABVN stimulation zone. The labeled neurons of the cuneate nucleus in a 1984 report are potentially related to the facial nerve fiber contributions that hitchhike the vagus nerve; this will be considered in the following section. The sympathetic axons of the C1–3 spinal nerves also contribute to the superior cervical ganglion since they do not receive direct sympathetic contributions from the spinal cord like the other spinal nerves at T1 and below (Netter, 1999; von Lanz and Wachsmuth, 2003). In addition, the ABVN does not have its own somatosensory sensory nucleus nor any somatosensory fibers. Regardless of whether these somatosensory axons of the ABVN join to the glossopharyngeus or the vagus nerves, they terminate in the trigeminal nucleus in the brainstem (Rhoton et al., 1966; Heimer, 1995) and so should actually be considered trigeminal nerve axons; this will be discussed in the following sections.

### In the Mastoid (Temporal Bone)

Before splitting into the three main bundles described in the previous section, the ABVN passes through the mastoid (Tekdemir et al., 1998). In the human mastoid, the ABVN crosses the fallopian canal, where it forms two main branches (Gasser, 1957; Schuknecht, 1974; Lang, 1983; May, 2000; Nageris et al., 2000; Diamond et al., 2011; Watanabe et al., 2016; Mulazimoglu et al., 2017).

The first branch of the ABVN reportedly connects to the *chorda tympani* branch of *the* *facial nerve* and also receives innervation from the posterior dura mater of the cranial fossa. The other division of the first branch of the ABVN contributes to the *external auditory meatus* and *concha zone of the auricular skin*, where AVNS is performed (Gasser, 1957; Schuknecht, 1974; Lang, 1983; May, 2000; Nageris et al., 2000; Diamond et al., 2011; Watanabe et al., 2016; Mulazimoglu et al., 2017; Figure 1).

The second branch of the ABVN also joins to *the facial nerve* *via* *the posterior auricular nerve of the facial nerve* before leaving the skull *via* the stylomastoid foramen and contributes to the posterior aspect of the auricula and the adjacent skull region (Gasser, 1957; Schuknecht, 1974; Lang, 1983; May, 2000; Nageris et al., 2000; Diamond et al., 2011; Watanabe et al., 2016; Mulazimoglu et al., 2017; Figure 1).

The type of axons that project from the facial nerve to the ABVN and vice versa remain obscure. The contributing axons from the facial nerve may have a *motor, general sensory, special sensory*, or even a *parasympathetic origin*, as the parasympathetic component of the facial nerve (the intermediate nerve, nervus intermedius) also enters the facial canal. Additionally, the sympathetic axons that join to the ABVN at the level of the jugular foramen can distribute to the external ear canal, the concha zone of the auricular skin, and the posterior aspect of the ear *via* the ABVN branch that contributes to the posterior auricular branch of the facial nerve. If we consider the retrograde pathway of the ABVN branch that contributes to the posterior branch of the facial nerve, a particular bundle of the posterior auricular branch of facial nerve axons joins the ABVN after it enters the skull *via* the stylomastoid foramen. In this scenario, which cannot be excluded in the context of the existing literature, these axons may also elongate up to the nucleus cuneatus, which corresponds to the center of the neck and potentially the auricular muscles, labeled and activated after ABVN HRV injections or stimulations in fMRI studies (Tekdemir et al., 1998;Frangos et al., 2015).

Finally, *facial and vestibulocochlear nerves also exchange axons* with the facial canal: another factor that confounds the elucidation of the origins and content of the axons that join to the ABVN in the facial canal (Nageris et al., 2000; Özdoğmuş et al., 2004; Diamond et al., 2011).

In the context of these extensive connections, before exiting the stylomastoid foramen or distributing to the external ear canal and auricula, the complexity of the ABVN is already established on account of its numerous axonal origins. No data in the literature has hitherto revealed the extent to which axons are distributed to each of the ABVN branches. Hence, AVNS studies cannot exclude these neural networks and should consider these potential neural contributions in their designs and interpretations of outcomes.

### Out of the Stylomastoid Foramen and the External Ear

The detailed territory of the ABVN in the posterior aspect of the auricula has not been mapped in the literature. The potential territory of ABVN axons in the posterior auricular branch of the facial nerve might distribute to the auricular muscle zones stimulated by the posterior auricular branch of the facial nerve. If we consider retrograde transmission in the ABVN, the posterior auricular nerve of the facial nerve that innervates the intrinsic and extrinsic auricular muscles would be hijacking the ABVN after it enters the skull *via* the stylomastoid foramen. As stated in the previous section, this model can account for the previously described HRP of the cuneate nucleus following HRP injections into the ABVN.

**Figure 2:**
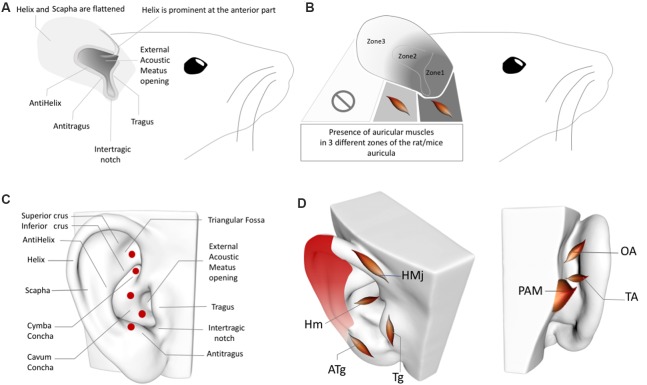
**(A)** Rat/Mouse Auricular Anatomy. **(B)** Rat/Mouse Auricular Muscle Zones. **(C)** Perforator artery zones of the human ear. **(D)** Inhomogeneous distribution of the perivascular sympathetic nerves on the human ear. Red gradient represents the density of perivascular innervation: Dark red: High density, Light red: Low density. OAM, Oblique auricular muscle; TAM, Transverse Auricular Muscle; PAM, Posterior Auricular Muscle; HMj, Helicis major muscle; Tg, Tragicus muscle; Hm, Helicis minor muscle; ATg, Anti-tragicus muscle. Adapted from Kiernan and Mitchell (1974), Chiu et al. (1979), Park and Roh (2002), Liugan et al. (2018), Cakmak et al. (2018) and Hong et al. (2019).

The existence of sympathetic axons that contribute to both the ABVN at the level of the jugular foramen and the posterior auricular branch of the facial nerve cannot be dismissed by current data. In this case, these sympathetic axons can distribute to the posterior aspect of the auricular skin *via* the posterior ABVN. In contrast, this distribution complicates interpretations of the AVNS because the sympathetic nerves also contribute to the perivascular innervation of the auricular arteries (Cakmak et al., 2018), and these auricular arteries have perforating branches that pierce the posterior aspect of the auricular cartilage (Figures 2C,D). These pass through to the internal aspect of the auricula and auricular skin, including the concha area where AVNS is performed (Park and Roh, 2002; Zilinsky et al., 2017; Cakmak et al., 2018). This perforating pathway provides not only a route for the sympathetic nerves to pass from the posterior surface of the ear to its anterior/interior surface but also a path for the parasympathetic axons from which the ABVN may originate. Our group recently demonstrated that the perivascular auricular innervation comprises sympathetic and cholinergic components in the human ear (Cakmak et al., 2018). The significance of this contribution and the inhomogeneity of these axons will be discussed in relation to the design and interpretations of ABVN studies in the following sections.

## Vagal Brainstem Nuclei Activation by Non-vagal Auricular Structures

In addition to the complexity of the axonal origins and contributions of ABVN, which must be considered in any analysis of ABVN stimulation, the interpretations of ABVN results in electrostimulation studies, including those that employ fMRI and neural tracing, feature unresolved ambiguities concerning auricular anatomy. This section will focus on the overlooked anatomical structures and non-vagal neural networks that can modulate vagal-related brainstem nuclei.

### Facial Nerve, Intrinsic Auricular Muscles, and Nucleus Cuneatus

The intrinsic auricular muscles of the human auricula and their innervation by the facial nerve axons are major anatomical and neural confounding factors of ABVN stimulation zones (Figure 2D). The helicis minor muscle, which is innervated by the facial nerve, is localized at the center of the ABVN zone used in numerous AVNS studies (Frangos et al., 2015). Similarly, the tragicus muscle is situated in the zone of the tragus, which is also used for AVNS investigations (Badran et al., 2018a,b). The other intrinsic muscles also constitute conflicting factors for the sham zones chosen in multiple studies. To date, none of the AVNS studies have considered the potential contribution of the intrinsic auricular muscles in the ABVN stimulation zones of the auricular muscles. Furthermore, the sham or control stimulation sites are also determined without this information (Frangos et al., 2015); consequently, while the active stimulation sites are muscle-free zones, the sham/control zones contain intrinsic auricular muscles and vice versa, preventing the proper interpretation of study results. Our group recently demonstrated that the stimulation of intrinsic auricular muscle zones improved the motor functions of patients with Parkinson’s disease (Cakmak et al., 2017). A recent study that placed active electrodes on the tragicus muscle zone claimed that the improvement of oromotor function occurs *via* the auricular vagus nerve (Badran et al., 2018b). Unfortunately, this unquestioned, biased approach is widespread among studies of the AVNS; indeed, investigations commonly reapply previous conceptions of the AVNS neural pathways that lack a basis in concise, accurate auricular anatomical knowledge.

Additionally, transcutaneous electrical stimulation features a parabolic electrical field that can induce the deeper stimulation of areas between electrodes. Median nerve stimulation of the wrist is a clear example of such a parabolic field effect: the median nerve situated at the core of the carpal tunnel zone of the wrist can be stimulated by transcutaneous electrical nerve stimulation electrodes placed 3 cm apart on the wrist (Urasaki et al., 1998; Ferretti et al., 2007; Maharjan et al., 2019). The structures, including intrinsic muscles and nerves located in the posterior aspect of the auricular muscles, can also potentially be stimulated by electrodes placed on the anterior surface of the auricula. To date, no study has investigated such a potential contribution, and it therefore cannot be ruled out.

The stimulation of the intrinsic muscles may influence multiple zones in the brainstem, including the nucleus cuneatus, cerebellum, and cortex. Selective muscle afferent nerve stimulation reportedly causes significant activation in motor-related areas relative to that induced by cutaneous stimuli (Wardman et al., 2014). Muscle afferent stimulation evokes more widespread cortical, subcortical, and cerebellar activations than do cutaneous afferents (Wardman et al., 2014). Separate pre- and post-central excitation foci were observed with muscle afferent stimulation, underscoring the importance of muscle afferent nerve stimulation in the modulation of cortical motor areas (Wardman et al., 2014).

Any ABVN stimulation should thus consider muscle-free zones in the ear to interpret the results related to the ABVN if the electrodes are placed on intrinsic auricular muscle zones. The nucleus cuneatus is a hub for neck muscles. As aforementioned, the posterior auricular branch of the facial nerve likely functions as the pathway to the nucleus cuneatus. A recent fMRI study also supported such a connection: stimulation of the antitragicus muscle zone (without considering the fact that this zone contains intrinsic auricular muscles) activated the nucleus cuneatus, whereas no activation in the nucleus cuneatus was observed when a non-muscular area of the ear was stimulated (Frangos et al., 2015). The nucleus cuneatus is also labeled when neurotracers are injected into the ABVN (Maslany et al., 1991). In another fMRI study (Badran et al., 2018a), the researchers also used the tragicus muscle zone as the AVNS zone, and the control zone was a muscle-free zone in the ear lobe. It was found that the supplementary motor area was only activated in the tragicus muscle zone stimulation, and this was postulated to be an outcome of AVNS. Any theories that explain the activation of the cortical and subcortical motor areas including nucleus cuneatus with auricular stimulation cannot exclude the intrinsic auricular muscle; moreover, these muscles can account for the activation of the nucleus cuneatus rather than that of the vagus nerve.

### Trigeminal Brainstem Nuclei

The nucleus ambiguus and the dorsal motor nucleus of the vagus are the two motor brainstem nuclei of the vagus nerve that contribute to striated and cardiac muscle rhythm and smooth and cardiac muscle contractibility, respectively (Geis and Wurster, 1980; Wang et al., 2001; Farmer et al., 2016), while the NTS is the viscerosensory nucleus. As stated in the Introduction, the vagus itself does not receive projections from or terminate in the somatosensory brainstem nuclei and instead conveys its somatosensory information to the brainstem nuclei of the trigeminal nerve (Rhoton et al., 1966; Heimer, 1995). The classification of the nuclei of the brainstem cranial nerve and the axonal bundles that compose cranial nerves is complex and likely inaccurate in many respects. The vagus conveys somatosensory information to the trigeminal nuclei *via* the ABVN, and this bundle is still considered the vagus nerve. In contrast, numerous hijacking axonal bundles in the cranial nerve system have not been classified in the same manner. The parasympathetic axons of the glossopharyngeal nerve hijack the auriculotemporal branch of the trigeminal nerve, and the parasympathetic fibers of the facial nerve also hijack the lingual, zygomatic, and lacrimal branches of the trigeminal nerve; however, these are still classified as being components of the glossopharyngeus and the facial nerves, respectively, because these axons terminate at, or their cell bodies are located in, the parasympathetic brainstem nuclei of the facial and glossopharyngeus nerves but not in the trigeminal brainstem nuclei. Similarly in terms of organization, the facial, glossopharyngeus, and vagus nerves do not have their own sensory brainstem nuclei but convey their somatosensory information to the brainstem nuclei of the trigeminal nerve—i.e., their somatosensory axons terminate in the trigeminal brainstem nuclei. However, these sensory axons are not classified under the trigeminal nerve system but are instead attributed to the ABVN. It can therefore be argued that the facial, glossopharygeus, and vagus nerves are actually hijacked by trigeminal nerve sensory fibers, which actually belong to the trigeminal nerve and not to the facial, glossopharyngeus, or vagus nerves. This line of reasoning indicates that they have traditionally been misclassified. Hence, ABVN stimulation can activate sensory nuclei of the trigeminal nerve not because of the axons of the ABVN itself, but rather because the trigeminal nerve axons hijack the ABVN.

Even in the case of a conventional, conservative argument that insists on the traditional but conflicting classification of these sensory fibers of the ABVN without considering their axonal origins, there is also another distinct neural route of trigeminal nerve sensory axons that project to the ABVN zone in the auricula that is independent of the ABVN route. The auriculotemporal nerve of the mandibular division of the trigeminal nerve contributes to the anterosuperior aspect of the auricula and supplies the skin over the tragus and helical crus regions of the auricula. This area also comprises the non-invasive auricular stimulation zone in numerous studies (Badran et al., 2018a,b; Niamtu, 2018). Further, the auriculotemporal nerve was reportedly found in 80% of the crus helicis. Interestingly, a gross auricular surface dissection study of 14 cadavers observed that 20% of the ABVN branches contribute to this zone (Peuker and Filler, 2002). Stimulation of these zones can also stimulate the trigeminal nuclei and the NTS *via* the auriculotemporal nerve as a distinct pathway independent from the ABVN (Figure 1).

It is also worth noting the limitations of Peuker and Filler (2002) in the context of overlooked neural networks because the study has been used as an auricular vagal nerve territory guide in numerous AVNS studies. In this gross dissection of 14 cadavers, the authors did not consider the ABVN division that contributes to the posterior auricular branch of the facial nerve. Moreover, no immunohistochemical mapping of the distal nerve territories was performed, but dissection-based recognition of the nerve bundles was considered for the proposed territories (Peuker and Filler, 2002). In addition, their investigation did not consider the perivascular cholinergic innervation of the human ear (Cakmak et al., 2018). In summary, the results concerning the vagus nerve territory are relatively incomplete and should not therefore be used as an absolute proxy for AVNS study design or interpretations.

### Problems With Taking NTS Activation as Proof of ABVN Stimulation

Labeling or activation in the viscerosensory nucleus of the NTS was advanced as a proof-of-concept for previous ABVN stimulation studies (Frangos et al., 2015). However, there are numerous issues and controversies regarding this monolog perspective. Numerous studies have clearly documented that the NTS can be directly and indirectly activated by multiple neural areas, most of which are present in the ABVN zone; this indicates that it is part of a brainstem reflex arc independent of the vagus nerve. In this section, we will focus on non-vagal pathways that can stimulate the NTS *via* AVNS zones.

Trigeminal nerve stimulation is one of the alternative pathways (DeGiorgio et al., 2003; Jean, 2008; Schrader et al., 2011) that can stimulate the NTS *via* its sensory nuclei. Stimulation of the ABVN zone, which overlaps with the trigeminal nerve, can also excite the trigeminal nerve (sensory axons within the ABVN or independent pathways). This may result in the activation of the trigeminal sensory nuclei of the brainstem, and then, as a reflex arc, the NTS can be stimulated (DeGiorgio et al., 2003; Jean, 2008; Schrader et al., 2011; Figure 1).

The facial nerve innervates the submandibular and sublingual glands with its parasympathetic component, which is carried by the chorda tympani branch of the facial nerve (Segal et al., 1996; Figure 1). The chorda tympani not only carries the parasympathetic axons of the facial nerve but also special sensory fibers with taste information from the anterior third aspect of the tongue. This special taste sensation relays to the rostral pole of the NTS, which also includes viscerotopography that represents visceral organs at its middle and lower poles (Altschuler et al., 1989, 1991; Broussard and Altschuler, 2000; Lemon and Di Lorenzo, 2002; Reddaway et al., 2012). While the axonal connections between the chorda tympani and the ABVN have also been demonstrated (Gasser, 1957; Schuknecht, 1974; Lang, 1983; May, 2000; Nageris et al., 2000; Diamond et al., 2011; Watanabe et al., 2016; Mulazimoglu et al., 2017), the origin and end terminals of these axons in the brainstem nuclei have yet to be investigated. As the facial nerve axons contribute to the ABVN in the facial canal, a portion of the ABVN axons might relay to the NTS *via* the facial nerve or axons of the facial nerve that hijack the ABVN in the temporal bone. In this context, the elucidation of the viscerotopical representation of the activated zones in the NTS is warranted to clarify the neural route to NTS and identify the axons that play a role in NTS stimulation in AVNS studies.

As emphasized previously, *the facial and vestibular nerves exchange axons* in the facial canal, and considering the axon exchange between the ABVN and the facial nerve, the exchanged axons between the facial and vestibular nerves might originate in the ABVN. Interestingly, it has been shown that the vestibular nerve can also stimulate the NTS (Yates et al., 1994; Figure 1).

Finally, several independent studies have shown that the stimulation of the peripheral spinal nerves in the hindlimb and forelimb modulates NTS activity (Noguchi and Hayashi, 1996; Tada et al., 2003; Imai et al., 2008; Wang et al., 2015). The underlying neural networks for this activation have yet to be elucidated, but the two spinal nerves (C2–C3) in the ABVN zone that innervate auricular skin feature well-known neural networks that can interact with the NTS (Figure 1). This neural network will be discussed together with sympathetic pathways in the next section.

In conclusion, NTS activation itself cannot be used as absolute proof of ABVN stimulation, and studies considering the clinical or physiological outputs of auricular stimulation should consider the trigeminal, facial, and other spinal nerves (including C2/C3 and their sympathetic nerves) in this zone to ascertain their sole or combined mechanisms of action in the auricular stimulation zone known as the ABVN.

As a final consideration in this discussion regarding the facial nerve and ABVN axonal exchanges, the motor nuclei of the cranial nerves have also been shown to contain cholinergic neurons, and the facial nerve motor nucleus has been reported to be one of the densest concentrations of cholinergic neurons of all the cranial nerve motor nuclei (Li et al., 2018).

### Locus Coeruleus (LC) Activation and Sympathetic Nerves in the Auricula

In addition to the aforementioned neural pathways that may be implicated in the mechanism of action of AVNS, the sympathetic nerves in the ABVN zone can also stimulate the NTS. Activity in the bilateral locus coeruleus (LC) observed with c-fos labeling or fMRI is also attributed to the stimulation of the ABVN and to NTS projections to the LC (Frangos et al., 2015). However, the LC also projects to the NTS, indicating an alternative or opposing mechanism of action. A major input to the LC originates from the nucleus paragigantocellularis (nPGi) in the reticular formation (Ennis and Aston-Jones, 1988; Kessel et al., 2008; Figure 1), which receives direct inputs from sympathetic neurons (Ennis and Aston-Jones, 1988; Berntson et al., 1998; Aston-Jones et al., 1996). This pathway is involved in transmitting sympathetic control and status information between the nPGi and the LC (Garcia-Rill, 1991; Limousin et al., 1997; Figure 1).

The sympathetic nerves distribute to the AVNS zone of the ABVN from at least three different neural routes: (1) the perivascular sympathetic nerves situated in the human ear (Cakmak et al., 2018); (2) the C2–3 spinal nerves that receive sympathetic axons from the superior cervical ganglion (Netter, 1999; von Lanz and Wachsmuth, 2003; Lingford-Hughes and Kalk, 2012); and (3) sympathetic axons that hijack the ABVN in the jugular foramen (Matthews and Robinson, 2014). All these sympathetic pathways can be stimulated within the zones of ABVN stimulation and may potentially stimulate the NTS *via* the LC. In the context of the described neural networks, the ABVN-NTS-LC axis cannot be postulated as the only explanation for the bilateral LC c-fos labeling after the stimulation of the cavum concha *via* AVNS. The cervical sympathetic ganglia should always be considered in the design and interpretation of AVNS studies as a potential labeling and/or a potential contributor of LC and NTS activation.

Therefore, the possible role of auricular sympathetic nerves and, consequently, their possible effects on AVNS study results cannot be excluded without the denervation of sympathetic nerves. It is worthwhile to note that the study by Shu et al. (2005) demonstrated that the antiepileptic effects of the auricular electrostimulation disappeared if the great auricular nerve (C2–C3) of a rat model of seizure was severed before electrical stimulation of the auricula.

A contrary argument is that activations of neither the LC or the NTS have been supported by control/sham region stimulations in AVNS studies (including human), and, hence, only stimulations in the active ABVN zone that includes the cymba concha may be postulated. This argument is addressed in the following section through discussion of the inhomogeneous distribution of the sympathetic nerves in the auricula.

## Auricular Anatomy: Inhomogeneity, Unique Cortical Representation, and Interspecies Differences

### Significant Confounding Effects of Inhomogeneity Problems

To date, the homogeneous distribution of sympathetic axons on the auricular skin has not been comprehensively investigated. Only a recent study on human ears has considered this topic. In a detailed histological and immunohistochemical labeling study, we demonstrated that perivascular sympathetic neurotransmitters are denser in the upper auricular zones adjacent to the cymba concha (Cakmak et al., 2018). This dense sympathetic labeling was not observed in the lower sections of the human auricula (Cakmak et al., 2018), and such an inhomogeneity should be considered in the design and interpretation of AVNS studies because most control/sham electrodes are placed in the lower aspects of the auricula. Therefore, if the control/sham stimulation is applied to the lower aspects of the auricula distant from the cymba concha, the potential confounding or otherwise significant role of auricular sympathetic nerves on AVNS study results cannot be excluded.

This problem may be overcome by preferring the upper levels of the auricula when applying electrodes in the sham/control groups and keeping the electrodes in the same auricular density zone of the sympathetic fibers, as in the case of vagal stimulation electrodes.

In addition to the inhomogeneous distribution of the sympathetic nerves in the human ear (Cakmak et al., 2018), perforating auricular arteries are also worth considering when placing electrodes in sham/control groups. Two independent group studies demonstrated that the posterior auricular artery—i.e., the periarterial sympathetic and cholinergic nerves—perforates the auricular cartilage in up to five locations (Figure 2C) to emerge from the posterior auricular surface at the anterior auricular surface, where they anastomose with branches of the superior temporal artery (Park and Roh, 2002; Zilinsky et al., 2017); four of the five perforated zones are reported in both of these studies (the perforating artery location in the triangular fossa was documented in only of the studies; Park and Roh, 2002; Zilinsky et al., 2017).

The auricular skin zones, which house these perforating arteries, likely contain a higher density of perivascular sympathetic axon bundles. The perforating arteries also distribute to the auricular skin areas that include the AVNS zones (Figure 2C), including the zone in the cymba concha where most AVNS studies place their active electrodes. It has also been reported that the perforator at the root of the helix consistently supplies the cymba and cavum of the concha as well as the posterior wall of the external auditory meatus, which fits perfectly with the AVNS zone (Park and Roh, 2002; Zilinsky et al., 2017). Hence, arteries—as well as perivascular sympathetic and cholinergic innervation—should be considered when determining the sham/placebo zones in the human ear. The concha zone, which corresponds with the perforating auricular artery (Figure 2C), as been demonstrated in two independent anatomical studies (Park and Roh, 2002; Zilinsky et al., 2017), is also postulated as the most effective zone in the context of fMRI activations. The confounding factor of the perforating artery in this zone of the ear is also underestimated and is not mentioned in the study report (Yakunina et al., 2017). In addition, all of the control stimulation zones in the same fMRI study were located lower to the concha stimulation zone, which cannot exclude the fact that a higher density of perivascular autonomic innervation exists in the upper sections of the human auricula (Cakmak et al., 2018; Figure 2D). To the best of our knowledge, our recent auricular stimulation study in patients with Parkinson’s disease is the only human study to uses a sham zone located in the high-density perivascular sympathetic auricular skin area, outside the perforation zone of the arteries and a zone without auricular muscles (Cakmak et al., 2017).

In addition to the documented inhomogeneous distribution of sympathetic fibers in the human ear, the territories of the great auricular nerve, which for the most part originates in C3 with contributions from C2 (Becelli et al., 2014), and of the lesser occipital nerve, which mainly originates in C2 with contributions from C3 (Waxenbaum and Bordoni, 2019), reportedly distributes differentially across the cymba concha area (the AVNS zone) among humans. It has further been demonstrated that the cymba concha is innervated by the lesser occipital nerve in a quarter (26%) of participants, which indicates that the same proportion of the experimental groups of AVNS studies may exhibit inhomogeneity (Pantaloni and Sullivan, 2000).

In summary, the active/sham/control AVNS zones should be carefully chosen according to documented sympathetic inhomogeneity, which is denser in upper zones, and perforating arteries in the human ear to overcome the potential influence of sympathetic nerves on study findings. In addition, within-study designs should be preferred to overcome documented anatomical variations of C2/C3 in the cymba concha area, which can influence a quarter of the results.

### The Unique Cortical Representation of the Auricula (External Ear)

In the final section, I would like to underscore the unique representation of the human ear in the cortex, which should be considered when interpreting cortical activations induced by auricular stimulation.

The representation of the human skin in the somatosensorial cortex is organized such that each region corresponds to one specific part of the human body (e.g., one zone of the somatosensorial cortex represents the hand only, while another zone represents the arm, and so on). In contrast, imaging studies have revealed that the cortical representation of the auricula is not as aligned as for the other parts of the human body. The auricula is represented by numerous different cortical zones that are distributed over the face, head, and neck representation areas (Nihashi et al., 2001) in the somatosensory cortex. Each of these areas belonging to auricular representation is segregated from the others, which means that they are not continuous across the somatosensorial cortex. It has been demonstrated that peripheral nerve stimulation can normalize the distortion of somatotopical representation in the cortex induced by pain conditions, including carpal tunnel and osteoarthritis, when it is applied through the nerves around the pathological condition zone (e.g., the forearm and wrist in carpal tunnel; Chen et al., 2003; Dhond et al., 2008). In this context, any study that performs AVNS for head and neck pain should also consider the unique cortical representation of the ear and other contributions of the sensory pathways that indicated in previous sections to explain the mechanism of action as well as the potential plasticity (normalization) of the somatosensorial representation of the head and neck *via* somatosensory stimulation.

### Interspecies Differences of the Auricula: Rat vs. Human

Furthermore, rat and human AVNS studies cannot be comparable in the context of the auricular muscle anatomy. While the rat auricula exhibits skin, cartilage, and auricular muscles, the muscle tissue is present only in the proximal segments, not in the distal third (Figures 2A,B). In the proximal two-thirds of the pinna, the dorsal auricular surface overlies muscular tissue, and in the proximal one-third, the external auricular muscles contribute to the dorsal aspect of the auricula, while the intrinsic muscles distribute at both surfaces of the auricular cartilage. In addition, the intrinsic muscles of the rat ear do not correspond with the intrinsic auricular muscles in humans (Figures 2B,D; Kiernan and Mitchell, 1974). In this context, any AVNS study of humans or rats should consider interspecies differences in the locations of intrinsic and extrinsic auricular muscles before choosing sham and control zones of the AVNS as well as when interpreting the results. Moreover, the localization of the perforating branches and inhomogeneity of the sympathetic distribution in the rat ear also require clarification in future research.

Some researchers use heart rate variability (HRV) to obtain evidence and/or monitor the AVNS (Badran et al., 2018b). The HRV can be altered by the modulation of the NTS and LC (Ellis and Thayer, 2010), and these brainstem nuclei are easily stimulated by other non-vagal neural networks—as explained in detail in the previous sections and Figure 1—in the AVNS zone. In addition, HRV alterations with auricular stimulation cannot be postulated as the mechanism of action of improved functioning/symptoms without excluding the contribution of other neural pathways. The auricula has extensive neural contributions, and it is not unusual for AVNS studies to stimulate multiple neural networks that can all function as key players in the mechanism of action.

In conclusion, AVNS studies (or better to refer to it as auricular electrical stimulation) are a promising avenue by which to explore numerous medical conditions; however, the lack of anatomical knowledge and inadequate study design in the context of a monolog anatomical concept impose clear limitations that may result in the misinterpretation of the mechanism of action. The present article helps to elucidate obscure neuroanatomical aspects of the ABVN and provide a deeper understanding of the auricular anatomy and the multiple neural networks that may influence its mechanism of action, thus helping to inform improved design of AVNS studies and the interpretations of their findings by considering all aspects of the auricular anatomy.

## Author Contributions

The author confirms being the sole contributor of this work and has approved it for publication.

## Conflict of Interest

The author declares that the research was conducted in the absence of any commercial or financial relationships that could be construed as a potential conflict of interest.
